# Adaptive Guided Filtering and Spectral-Entropy-Based Non-Uniformity Correction for High-Resolution Infrared Line-Scan Images

**DOI:** 10.3390/s25144287

**Published:** 2025-07-09

**Authors:** Mingsheng Huang, Yanghang Zhu, Qingwu Duan, Yaohua Zhu, Jingyu Jiang, Yong Zhang

**Affiliations:** 1 University of Chinese Academy of Sciences, Beijing 100049, China; huangmingsheng@mail.ustc.edu.cn (M.H.); zhuyanghang22@mails.ucas.ac.cn (Y.Z.); zhuyaohua@mail.sitp.ac.cn (Y.Z.); jiangjingyu23@mails.ucas.ac.cn (J.J.); 2 Shanghai Institute of Technical Physics, Chinese Academy of Sciences, Shanghai 200083, China; duanqw2023@shanghaitech.edu.cn; 3 School of Information Science and Technology, ShanghaiTech University, Shanghai 201210, China

**Keywords:** non-uniformity correction, infrared line-scan imaging, adaptive guided filtering, spectral entropy suppression, high-resolution imaging

## Abstract

Stripe noise along the scanning direction significantly degrades the quality of high-resolution infrared line-scan images and impairs downstream tasks such as target detection and radiometric analysis. This paper presents a lightweight, single-frame, reference-free non-uniformity correction (NUC) method tailored for such images. The proposed approach enhances the directionality of stripe noise by projecting the 2D image into a 1D row-mean signal, followed by adaptive guided filtering driven by local median absolute deviation (MAD) to ensure spatial adaptivity and structure preservation. A spectral-entropy-constrained frequency-domain masking strategy is further introduced to suppress periodic and non-periodic interference. Extensive experiments on simulated and real datasets demonstrate that the method consistently outperforms six state-of-the-art algorithms across multiple metrics while maintaining the fastest runtime. The proposed method is highly suitable for real-time deployment in airborne, satellite-based, and embedded infrared imaging systems. It provides a robust and interpretable framework for future infrared enhancement tasks.

## 1. Introduction

### 1.1. Background

With advancements in infrared detection technologies, scanning imaging systems, and cooling materials, infrared imaging has wide applications in military reconnaissance [1], aerospace remote sensing [2,3], night-vision surveillance [4,5], and industrial inspection [6,7]. However, line-scan infrared systems inherently suffer from non-uniformity noise due to cumulative effects such as detector integration drift, temperature variation, electronic readout instability, and scene-dependent radiation fluctuations [8,9,10,11]. These factors often produce structured horizontal stripe noise along the scanning direction, significantly degrading image quality and impacting downstream tasks such as target detection [12,13,14], edge extraction [15], and temperature inversion [16,17].

### 1.2. Related Work

Non-uniformity correction (NUC) methods for infrared images are generally classified into reference-based [18] and scene-based approaches [19,20,21]. Reference-based methods rely on external standard targets, such as blackbody sources and temperature-controlled references [22], and typically calibrate the system using two-point [23,24] or multi-point response models [25]. While these methods achieve high accuracy in laboratory settings and are suitable for system initialization or periodic recalibration, their strong dependence on external references limits their applicability in real-time and adaptive processing scenarios [26,27,28].

In contrast, scene-based methods are more flexible and practical. They utilize the image’s structural, statistical, or temporal characteristics to estimate non-uniformity noise. These techniques have gained increasing attention recently due to their real-time adaptability [29]. Based on modeling strategies, scene-based NUC methods can be divided into four main types:

(1) Filtering-based methods: These extract low-frequency bias components using spatial or frequency domain techniques such as mean [30], Gaussian [31], or guided filtering [32,33,34]. The estimated noise is subtracted from the original image. Although computationally efficient, these methods often lead to over-smoothing or texture loss in regions with varying stripe intensities or complex backgrounds [35,36,37,38].
(2) Statistical modeling methods: These use statistical priors such as brightness distributions, row/column means, or local statistics to estimate noise [39]. While training-free and straightforward, their adaptability to non-stationary noise is limited [40,41].
(3) Model optimization methods: These construct priors and regularization terms using approaches like total variation [42,43], wavelet transforms [44], curvelet transforms [45], or low-rank decomposition [46]. Although capable of preserving details and separating structured noise, they often suffer from high computational costs and sensitivity to parameter tuning [47,48,49].
(4) Neural-network-based methods: End-to-end learning models [50], including convolutional neural networks [51], residual networks [52], and autoencoders [53], have shown strong performance on labeled datasets. However, their reliance on extensive training data, poor generalization to unseen scenes, and limited interpretability constrain their practical deployment [54,55,56].

Recently, lightweight deep learning architectures—such as MobileNet-based UNet variants [57] or attention-enhanced residual networks [58]—have been proposed to mitigate the computational burden of full-scale CNNs. However, these models still require substantial offline training, lack robustness to unseen noise distributions, and remain challenging to interpret physically [59]. Moreover, their deployment on embedded or hardware-limited systems remains difficult due to memory and latency constraints. Therefore, a critical need remains for training-free, interpretable, and computationally efficient NUC algorithms suitable for real-time applications.

### 1.3. Our Contributions

To overcome existing limitations, an adaptive, single-frame non-uniformity correction (NUC) algorithm is proposed for high-resolution infrared line-scan images. Compared to our previous work using one-dimensional guided filtering and linear modeling [10], the proposed method introduces three main improvements: a row-mean projection strategy to highlight stripe directionality, an MAD-based adaptive smoothing scheme for better structural preservation, and a spectral-entropy-based frequency-masking mechanism for effective suppression of both periodic and aperiodic noise.

The main contributions of this work are summarized as follows:

(1) Row-mean-based 1D modeling: Projects 2D images into 1D sequences through row averaging, which improves stripe directionality, simplifies modeling, and boosts sensitivity to directional noise.
(2) MAD-driven adaptive guided filtering: A fusion framework combines global background trends with local structural features. Filter scales are adaptively chosen based on local median absolute deviation (MAD), allowing spatially adaptive smoothing while maintaining structural accuracy.
(3) Spectral-entropy-based frequency masking: A frequency-domain suppression method is introduced that uses spectral entropy to build adaptive thresholds, enabling the isolation and suppression of both periodic and aperiodic interference without requiring iterative optimization or high-order reconstruction.
(4) Lightweight and efficient implementation: The complete algorithm is streamlined for real-time applications. It requires only a single pass of guided filtering and two FFT operations, making it suitable for embedded and resource-constrained platforms.

Extensive experiments on synthetic and real infrared datasets demonstrate that the proposed method consistently outperforms state-of-the-art approaches in correction accuracy, robustness, and processing speed. These results indicate strong potential for real-time deployment in airborne, satellite, and industrial infrared imaging systems.

## 2. Materials and Methods

**Figure 1:**
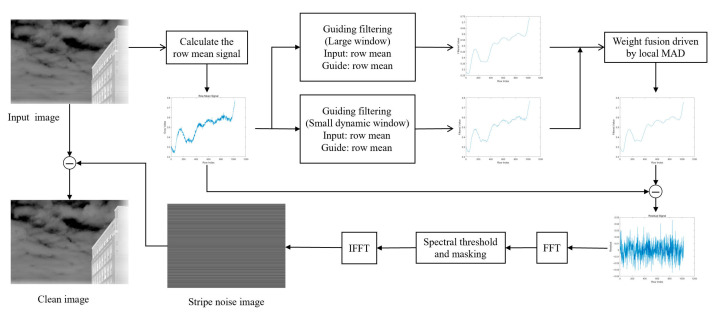
Overview of the proposed non-uniformity correction framework.

Figure 1 presents the overall workflow of the proposed algorithm, which consists of four main stages: (1) Row-mean signal modeling: The input 2D infrared image is averaged along each row to generate a 1D signal representing the stripe pattern along the horizontal direction [60]. (2) Multi-scale guided filtering and adaptive fusion: Guided filtering is performed at multiple preset spatial scales. A local median absolute deviation is calculated to determine adaptive weights. These weights fuse the filtered results and generate a 1D background estimation signal. (3) Frequency domain processing and stripe suppression: A one-dimensional Fourier transform is applied to the residual signal. Spectral entropy and global statistics are used to calculate an adaptive threshold. A frequency-domain mask is constructed to suppress dominant frequency components. (4) Stripe compensation and image restoration: The 1D filtered residual is replicated along the column direction to form a 2D correction map, subtracted from the original image to produce the corrected output.

**Figure 2:**
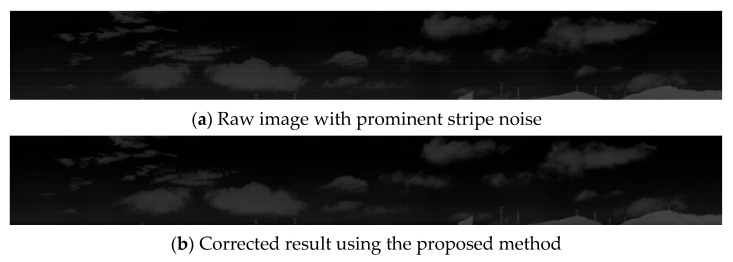
Correction results on real high-resolution infrared line-scan imagery.

Figure 2 presents the non-uniformity correction results of our method on real high-resolution infrared line-scan images.

### 2.1. One-Dimensional Modeling and Orientation Feature Extraction

Let the input infrared image be denoted as I(i,j), where i∈[1,H] and j∈[1,W] represent the row and column indices, respectively. The row-mean signal r(i) is calculated by averaging all pixel values along each row:

(1) $$r\left(i\right)=\frac{1}{w}\sum_{j=1}^{w}I\left(i,j\right)$$

**Figure 3:**
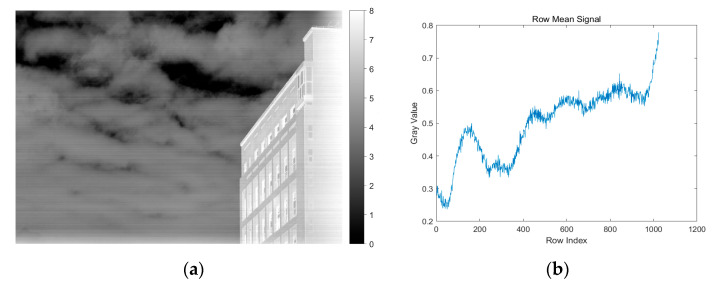
(**a**) Original infrared image with an 8-level gray-scale color bar; (**b**) row-mean signal.

The resulting one-dimensional signal $r(i)\in R^{H\times 1}$ represents the average radiation intensity across each scan line. Figure 3 shows the original input image and the corresponding row-mean signal plot, which highlights the stripe structure along the horizontal direction. This 1D signal is subsequently used as the input for the guided filtering process in the next stage.

### 2.2. Multi-Scale Guided Filtering with MAD-Based Adaptation

#### 2.2.1. Principle of Guided Filtering

Guided filtering is an edge-preserving image smoothing technique based on a local linear model, initially introduced by He [61]. Due to its efficiency and simplicity, it has been widely applied in image denoising, detail enhancement, and structure-preserving filtering. Unlike traditional mean or Gaussian filters, the guided filter incorporates structural information from a guidance image, producing smoothed results that better preserve edge boundaries. In the context of this work, both the input and guidance signals are set to the row-mean signal r(i).

Within a local window $w_{k},$ the guided filter assumes a linear relationship between the guidance signal $I_{i}$ and the filtering output $q_{i}$:

(2) $$q_{i}=a_{k}I_{i}+b_{k},\forall i\in \omega_{k}$$

where $a_{k}$ and $b_{k}$ are linear coefficients assumed to be constant within the window. These coefficients are estimated by minimizing the following cost function, which enforces fidelity to the input while preventing overfitting through regularization:

(3) $$E\left(a_{k},b_{k}\right)=\sum_{i\in w_{k}}\left({\left(a_{k}I_{i}+b_{k}-p_{i}\right)}^{2}+\varepsilon a_{k}^{2}\right)$$

where ε is a regularization parameter used to suppress $a_{k}$ from being too large to prevent overfitting; by solving the above minimization problem, the following can be obtained:

(4) $$a_{k}=\frac{\frac{1}{\left|w\right|}\Sigma_{i\in w_{k}}I_{i}p_{i}-\mu_{k}{\bar{p}}_{k}}{\sigma_{k}^{2}+\varepsilon }$$

(5) $$b_{k}={\bar{p}}_{k}-a_{k}\mu_{k}$$

where $\mu_{k}$ and $\sigma_{k}^{2}$ denote the mean and variance of the guidance signal $I_{i}$ within window $\omega_{k}$, and ${\bar{p}}_{k}$ is the mean of the input signal $p_{i}$ in the same window. The final output $q_{i}$ obtained by averaging the results of all overlapping windows that include pixel i:

(6) $$q_{i}=\frac{1}{\left|w\right|}\sum_{k|i\in w_{k}}\left(a_{k}I_{i}+b_{k}\right)$$

Assuming uniform window overlap and symmetric filtering, this simplifies to

(7) $$q_{i}={\bar{a}}_{i}I_{i}+\bar{b_{i}}$$

where ${\bar{a}}_{i}$ and $\bar{b_{i}}$ are the averaged coefficients over all windows containing pixel i. This formulation ensures that the output remains edge-aware and less noise-sensitive, which is particularly important for preserving directional structures in the subsequent fusion process.

#### 2.2.2. MAD-Driven Dynamic Fusion Strategy

To improve robustness to local intensity variations and stripe strength fluctuations, the global statistical characteristics of the row-mean signal r(i) are utilized to guide the smoothing scale. A multi-scale guided filtering strategy is employed, where the smoothing radius is dynamically adjusted based on the local MAD.

First, a large-window guided filter is applied to r(i) using a fixed radius $R_{large}$ and smoothing coefficient ϵ. The result is denoted as

(8) $$r_{large}\left(i\right)=GuideFilter\left(r(i),r(i),R_{large},\epsilon \right)$$

To enable adaptive smoothing across varying regions, additional filtered results are precomputed using a set of neighborhood radii $R\in \left[R_{min},R_{large}\right]\left\{r_{R}\left(i\right)\right\}$. For each index i, a local window centered at i is selected, and the MAD is computed as follows:

(9) $${MAD}_{local}\left(i\right)=median\left(\left|r(k)-median\left(r(k)\right)\right|\right),\;k\in \left[i-r,i+r\right]$$

Based on the ratio of the local MAD to the global MAD, the inverse proportionality strategy is used to adjust the local filtering radius, R(i), which is shown in Equation (10):

(10) $$R\left(i\right)=max\left(R_{min},min\left(R_{large},\left[R_{large}-\alpha \cdot \frac{{MAD}_{local}\left(i\right)}{{MAD}_{global}\left(i\right)+\delta }\right]\right)\right)$$

where α is the scaling factor, and δ is a small constant to avoid division by zero.

**Figure 4:**
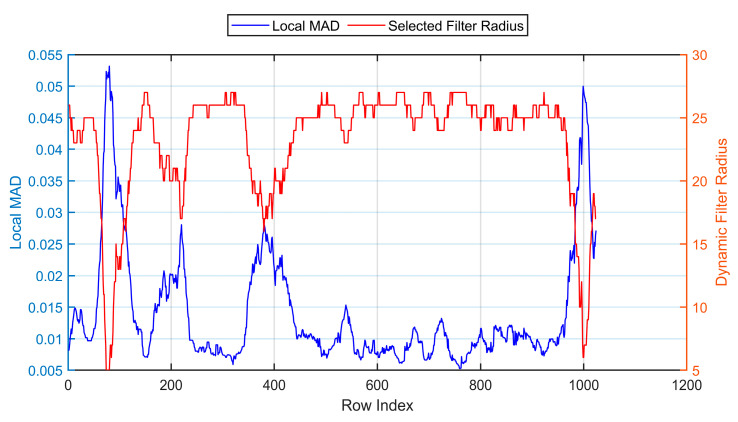
Variation of local MAD and corresponding dynamic filter radius.

As illustrated in Figure 4, the filtering radius determined by this strategy is significantly negatively correlated with the local MAD. The blue curve shows the variation of local MAD across image rows, while the red curve represents the corresponding dynamic filtering radius.

The adaptive filter result $r_{R(i)}\left(i\right)$ is then combined with the large-window result $r_{large}\left(i\right)$ through soft weighting:

(11) $$\omega \left(i\right)=\frac{1-\exp(-\alpha \cdot {MAD}_{local}(i))}{1-\exp(-\alpha \cdot {MAD}_{global}+\gamma )}$$

Finally, the background estimate for adaptive fusion $\hat{r}\left(i\right)$ is calculated as follows:

(12) $$\hat{r}\left(i\right)=\omega \left(i\right)\cdot r_{R\left(i\right)}\left(i\right)+(1-\omega (i))\cdot r_{large}(i)$$

The result $\hat{r}(i)$ preserves the overall trend of the row-mean signal while adapting to local structures and is used for residual computation in the subsequent frequency-domain stage.

**Figure 5:**
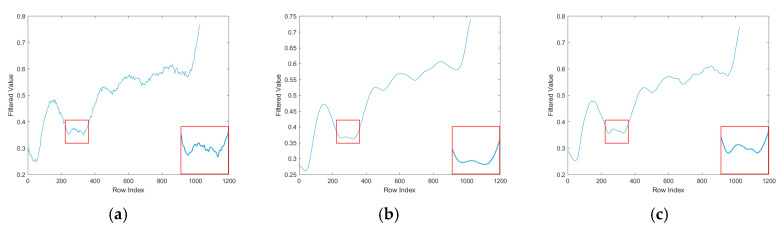
MAD-driven fusion strategy. (**a**) R = 5 small window filtering result; (**b**) R = 30 large window filtering result; (**c**) MAD-driven fusion result.

As can be seen in Figure 5, when the filtering radius R=5, the small-scale guided filtering in Figure 5a retains more noise and has more details but is not smooth enough. When the filter radius R=30, the large-scale guided filtering in Figure 5b is too smooth, and the stripes are suppressed, but the details are lost, and the smoothness and details of the image are better preserved by the MAD-driven fusion strategy, which is both robust and adaptive.

### 2.3. Frequency-Domain Spectral Entropy Gating Mechanism

A frequency-domain filtering scheme is introduced to further suppress the residual periodic stripe noise in the row-mean domain. The residual signal is first obtained by subtracting the smoothed background estimate $\hat{r}\left(i\right)$ from the original row-mean signal:

(13) $$\delta \left(i\right)=r\left(i\right)-\hat{r}\left(i\right)$$

The residual signal $\delta \left(i\right)$ is fast Fourier transformed to obtain the spectral expression:

(14) $$F\left(i\right)=F\left[\delta \left(i\right)\right]=A\left(i\right)\cdot {\mathrm{e}}^{j\phi \left(i\right)}$$

where $A\left(i\right)=\left|F\left(i\right)\right|$ is the amplitude spectrum, and $\phi \left(i\right)=arg\left(F\left(i\right)\right)$ is the phase spectrum. The spectral entropy H is introduced to measure the residual signal’s spectral complexity. The probability distribution of the normalized amplitude is defined as

(15) $$P\left(i\right)=\frac{A\left(i\right)}{\Sigma_{i}A\left(i\right)},H=-\sum_{i}P\left(i\right)\log\left(P\left(i\right)+\varepsilon \right)$$

where ε is a small constant to avoid numerical singularities. Spectral entropy reflects the energy dispersion and structural disorder in the frequency domain. Based on the spectral entropy, an adaptive threshold, T, for amplitude suppression, is defined as

(16) T=μ+β⋅H⋅σ

where μ=mean(A(i))σ=std(A(i)), and β is a scaling coefficient that controls the impact of entropy on the threshold.

A binary suppression mask is then constructed:

(17) $$\tilde{F}\left(i\right)=\left\{\begin{array}{c} 0\;\;\;\;\;\;\;\;\;\;\;\;\;,\;\;\;\;\;\;A\left(i\right)\geq T\\ F\left(i\right)\;\;\;\;\;\;\;,\;\;\;\;\;\;A\left(i\right)<T \end{array}\right.$$

Equation (17) zeros the frequency components above the threshold to avoid streak mains frequency leakage. The suppressed frequency component is further obtained:

(18) $$F_{removed}\left(i\right)=F\left(i\right)-\tilde{F}\left(i\right)$$

A Fourier inverse transform is performed on $F_{removed}\left(i\right)$ to obtain the corresponding time-domain stripe estimate:

(19) $$\tilde{\delta }\left(i\right)=F^{-1}\left[F_{removed}\left(i\right)\right]$$

The generated signal $\tilde{\delta }\left(i\right)$ represents the estimated streak noise component in the row-mean domain.

### 2.4. Stripe Expansion and Image Restoration

The estimated 1D residual noise signal $\tilde{\delta }\left(i\right)$ obtained through frequency-domain processing is expanded into a 2D spatial pattern for correction. This is achieved by replicating the 1D signal across all columns to construct a redundant 2D noise map:

(20) $$\Delta \left(i,j\right)=\tilde{\delta }\left(i\right),\forall j\in \left[1,W\right]$$

This map $\Delta \left(i,j\right)$ represents the estimated horizontal stripe interference and is subtracted from the original image to generate the corrected image:

(21) $$\hat{I}\left(i,j\right)=I\left(i,j\right)-\Delta \left(i,j\right)$$

**Figure 6:**
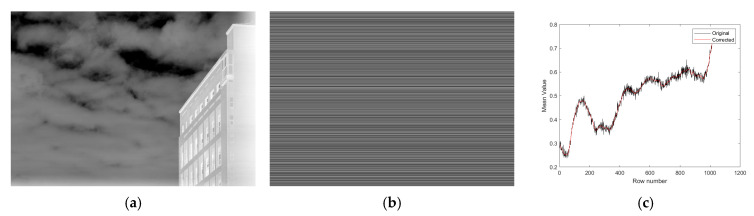
Image restoration results. (**a**) Corrected image; (**b**) streak noise map; (**c**) comparison of row-mean results.

Figure 6 illustrates the image restoration results. The estimated stripe noise pattern, generated by extending the 1D residual along the column direction, is shown in Figure 6b. The final corrected image, obtained by subtracting this pattern from the original input, is displayed in Figure 6a. Figure 6c compares the row-wise intensity curves before and after correction, demonstrating a significant reduction in horizontal non-uniformity.

### 2.5. Experimental Setup

The experiments are conducted on five datasets: OSU [62], KAIST [63], LLVIP [64], Tendero’s dataset [40], and a proprietary long-wave infrared weekly scanned line-scan dataset. All methods are executed under the same hardware and software environment. The computing platform includes a 12th-generation Intel Core i7-12700H CPU (Intel Corporation, Santa Clara, CA, USA) @ 3.61 GHz, 32 GB RAM, and a 64-bit Windows operating system. All algorithms are implemented and tested using MATLAB 2024a.

## 3. Results

To evaluate the non-uniformity correction performance of the proposed algorithm, we conduct a comparative study with several representative state-of-the-art methods developed in recent years for infrared detectors. These methods can be broadly categorized into four groups:

(1) Frequency-domain filtering-based methods: the two-stage filtering (TSF) approach proposed by Zeng in 2018 [36] combines frequency-domain filtering with one-dimensional row-guided filtering, aiming to remove stripe artifacts while preserving image structures.
(2) Spatial-domain guided filtering approaches: the guided filtering with linear fitting (GFLF) method proposed by Li in 2023 [32] performs non-uniformity correction via one-dimensional guided filtering and regression modeling. The ASNR method proposed by Hamadouche in 2024 [33] further integrates frequency mask extraction with guided filtering to enhance stripe suppression capability.
(3) Optimization-based methods: the ADOM model [49] incorporates Weighted Paradigm Regularization and a Momentum Update Mechanism within an ADMM optimization framework to correct non-uniformity artifacts adaptively.
(4) Traditional methods: the Median-Histogram-Equalization-based Non-uniformity Correction Algorithm (MIRE) proposed by Tendero in 2012 [40] and the Estimating Bias by Minimizing the Differences Between Neighboring Columns (MDBC) method introduced by Wang in 2016 [47] serve as classical baselines in non-uniformity correction, relying on histogram statistics and local column differences, respectively.

### 3.1. Noise Modeling and Analysis

Unlike conventional infrared focal plane arrays, where each pixel operates independently, line-scan infrared detectors utilize a single column of pixels that move vertically to scan the scene line by line. Since each image row is acquired by the same detector element, any gain drift or bias error in a specific pixel will manifest consistently across the corresponding image row. This results in structured horizontal stripe artifacts characteristic of line-scan infrared systems.

To model this non-uniformity, a hybrid gain–bias–white noise model is adopted, which can be expressed as

(22) $$I_{noise}\left(i,j\right)=g\left(i\right)\cdot I_{ideal}\left(i,j\right)+b\left(i\right)+n\left(i,j\right)$$

where $I_{noise}\left(i,j\right)$ denotes the observed image, $I_{ideal}\left(i,j\right)$ denotes the ideal noise-free image, $g\left(i\right)$ and $b\left(i\right)$ denote the gain term and bias term of the ith line, respectively, obeying the Gaussian distributions $g\left(i\right)\sim N\left(1,\sigma_{g}^{2}\right)$ and $b\left(i\right)\sim N\left(0,\sigma_{b}^{2}\right)$, and $n\left(i,j\right)\sim N\left(0,\sigma_{white}^{2}\right)$ is the zero-mean white noise.

In addition to these structured distortions, periodic noise is frequently observed in infrared imaging systems, typically caused by power supply instability, clock jitter, or electronic readout nonlinearities. This type of noise exhibits periodic oscillations along the column direction and is modeled as

(23) $$P\left(i,j\right)=A\cdot \cos\left(2\pi f_{0}j+\phi \right)$$

where A denotes the interference amplitude, $f_{0}$ is the normalized spatial frequency, and ϕ is the phase offset. Periodic noise appears as sharp peaks in the frequency domain, which differ from natural broadband signals. The proposed masking strategy removes structural stripes and filters periodic interference, improving overall robustness.

### 3.2. Evaluation Indicators

This study employs a combination of reference-based and no-reference evaluation metrics to comprehensively assess the effectiveness of non-uniformity correction. These metrics evaluate the algorithm’s performance, including restoration quality, structure preservation, and stripe suppression. The reference-based metrics include peak signal-to-noise ratio (PSNR), roughness, and structural similarity index (SSIM). In contrast, the no-reference metrics include gradient consistency (GC) and frequency-domain noise ratio (NR).

PSNR quantifies image quality by evaluating the mean squared error (MSE) between the restored image and a reference. A more excellent PSNR value typically reflects higher reconstruction accuracy. It is defined as

(24) $$PSNR=10\log_{10}\left(\frac{MAX^{2}}{MSE}\right)$$

where MAX represents the maximum pixel intensity allowable in the image.

Roughness assesses spatial intensity variation and helps identify residual artifacts. It is calculated from the cumulative gradient magnitude in horizontal and vertical directions. To evaluate structural consistency, the roughness of the processed image is compared to that of the original. The closer these values are, the more consistent the image structure is. The formula is given by

(25) $$R=\frac{\sum \left|\nabla_{x}I\left|+\Sigma \right|\nabla_{y}I\right|}{\sum \left|I\right|}$$

where $\nabla_{x}=\left[-1,1\right]\nabla_{y}=\left[-1,1\right]$ are first order difference operators, and I is the input image.

SSIM evaluates structural similarity between the corrected image and the reference, focusing on brightness, contrast, and texture. A value closer to 1 indicates higher structural consistency. SSIM is computed as

(26) $$SSIM=\frac{\left(2\mu_{\hat{I}}\mu_{I}+C_{1}\right)\left(2\sigma_{\hat{I}I}+C_{2}\right)}{\left(\mu_{\hat{I}}^{2}+\mu_{I}^{2}+C_{1}\right)\left(\sigma_{\hat{I}}^{2}+\sigma_{I}^{2}+C_{2}\right)}$$

The symbols $\mu_{I}$, $\mu_{\hat{I}}$, $\sigma_{I}^{2},$ and $\sigma_{\hat{I}}^{2}$ represent the mean and variance of images I and $\hat{I}$, respectively. The term $\sigma_{\hat{I}I}$ corresponds to the covariance between the two images. Constants $C_{1}$ and $C_{2}$ are introduced to avoid instability caused by small denominators.

The GC metric quantifies differences in gradient strength between the enhanced image and the original one affected by noise. A lower GC score implies better structural preservation. It is defined as

(27) $$GC=\frac{\Sigma \left|\nabla I_{n}-\nabla I_{c}\right|}{\Sigma \left|\nabla I_{n}\right|}$$

where $I_{n}$ is the noise image, $I_{c}$ is the corrected image, and ∇ is the gradient.

NR evaluates the change in spectral energy caused by noise suppression. It reflects the level of frequency-domain filtering. A higher NR indicates more effective noise removal. The metric is computed as

(28) $$NR=\frac{{\left\|F\left(I_{n}\right)\right\|}^{2}}{{\left\|F\left(I_{c}\right)\right\|}^{2}}$$

where $F\left(\cdot \right)$ denotes the 2D Fourier transform, $I_{n}$ is the noise image, and $I_{c}$ is the corrected image.

### 3.3. Ablation Experiments

Five experimental configurations are designed to evaluate each core sub-module’s contribution to the overall performance of non-uniformity correction, as shown in Table 1. The tested modules include DR (row-mean dynamic radius guided filtering), FM (frequency-domain mask filtering), and ET (spectral-entropy-based adaptive thresholding). The baseline model, B0, uses only large-window guided filtering without any adaptive components. B1 adds the DR module to assess the effect of local radius adaptation. B2 incorporates only the FM module to verify its ability to suppress periodic stripe noise. B3 combines DR and FM to examine their complementarity. Finally, B4 represents the complete model, integrating all three modules.

In these experiments, except for the tested modules, other parameters are fixed: the large-window guided filter radius is set to 30, the smoothing coefficient ε=0.16, and the spectral entropy threshold scaling factor β=0.003. All evaluations are conducted on real high-resolution infrared line-scan images. The original image size is 1024 × 55,000, and a 1024 × 1434 region is used for testing.

The quantitative results of the five configurations are presented in Table 2. Compared with the baseline B0, adding DR in B1 improves PSNR and SSIM, indicating enhanced restoration and structural consistency. However, the roughness value remains far from the original image’s 0.036, suggesting that DR alone has limited ability to suppress horizontal undulating noise. GC is notably reduced in B2, where FM is used alone, confirming that frequency-domain masking effectively attenuates periodic interference. Yet, the roughness increases to 0.027, implying a risk of over-smoothing. When DR and FM are jointly applied in B3, all metrics show noticeable improvement, indicating that the combination achieves a better trade-off between suppression and structure preservation. With the addition of the ET module, the whole model B4 achieves the best performance across all five metrics. Its roughness and NR values are closest to the original image, demonstrating that entropy-based thresholding improves robustness while avoiding excessive smoothing.

**Figure 7:**
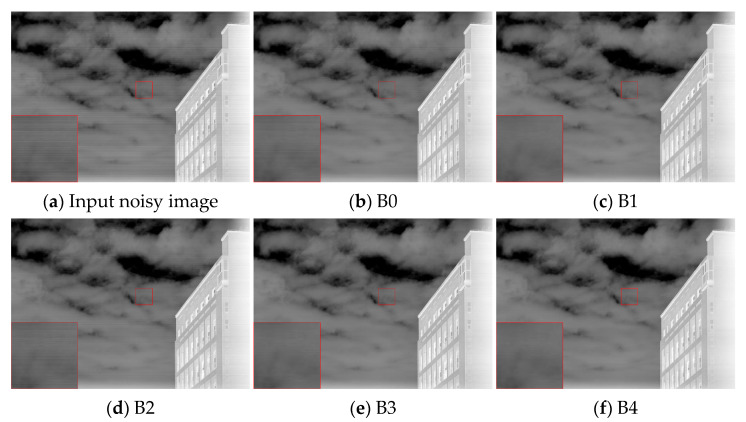
Visual comparison of correction results under different module combinations.

Figure 7 provides a visual comparison of the different configurations. B0 exhibits prominent horizontal stripes. B1 and B2 partially suppress noise, leaving residual patterns or smoothing artifacts. B3 improves the clarity of cloud structures and building edges. In contrast, B4 eliminates structural and periodic artifacts and more effectively restores natural details. These visual observations align well with the quantitative trends in GC and roughness, further confirming the role of the ET module in balancing detail preservation and noise suppression.

### 3.4. Parameter Sensitivity Analysis

To evaluate the robustness of the proposed algorithm concerning key hyperparameters, we conduct a sensitivity analysis on two categories of representative parameters in the spatial and frequency domains. Three hyperparameters are tested: (1) large window guided filter radius r, (2) guided filter smoothing coefficient ϵ, (3) spectral entropy threshold scaling factor β.

We select images from the KAIST dataset and systematically vary each parameter while keeping the others at their default values. Noise is added based on the gain–bias model defined in Equation (22), where the gain $g\left(i\right)\sim N\left(0,0.02\right)$ and $b\left(i\right)\sim N\left(0,0.02\right)$, with no additional white noise included.

**Figure 8:**
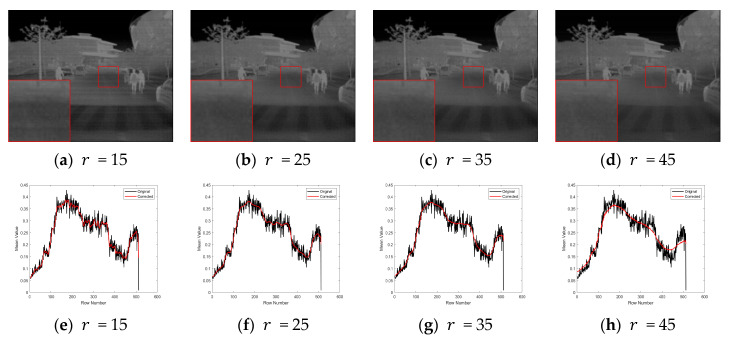
Sensitivity to large-window radius r.

Figure 8 shows the effect of varying the large-window radius r. When r=15, the corrected image still exhibits high-frequency jitter, and residual stripe noise remains visible. As the radius increases to 25–35, the streaks are effectively suppressed, and local contrast is well maintained. However, when r=45, the image becomes over-smoothed, and fine details in low-contrast regions are noticeably attenuated. Therefore, a recommended range for r is between 25 and 35.

**Figure 9:**
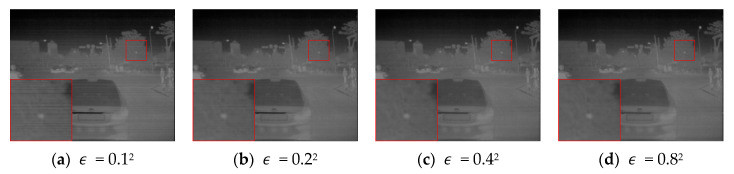
Sensitivity to smoothing coefficient ε.

Figure 9 presents the results under different smoothing coefficients ϵ; when ϵ = 0.12 or ϵ = 0.22, stripe noise is still visible in the red box region. Increasing ϵ = 0.42 improves suppression while preserving edge details. However, at ϵ = 0.82, excessive smoothing occurs, weakening vehicle contours and road textures. Thus, a moderate ϵ value offers a good trade-off between smoothing and detail preservation.

**Figure 10:**
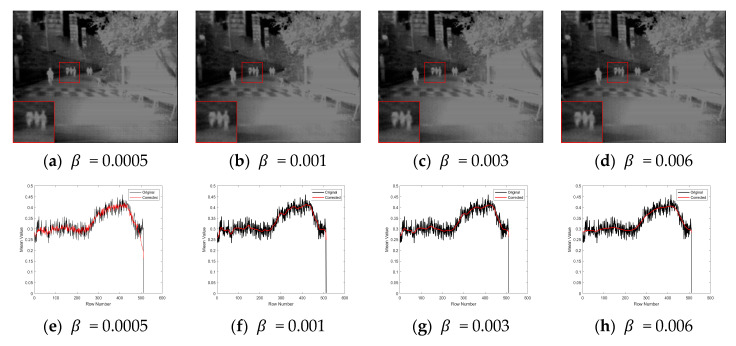
Sensitivity to spectral threshold β.

Figure 10 evaluates the influence of the spectral threshold coefficient β. As it increases, the adaptive threshold rises, which enhances stripe suppression by treating more frequency components as noise; however, an excessively large value may lead to over-smoothing, blurring edge structures, and flattening row-mean profiles. In contrast, spectral suppression is insufficient if β is too small, and residual noise remains. Considering these effects, we recommend setting β ∈ [0.001, 0.003] for optimal performance.

### 3.5. Method Performance Comparison and Analysis

#### 3.5.1. Comparison Algorithm and Experimental Setup

We compare our algorithm with six state-of-the-art methods: MIRE [40], MDBC [47], TSF [36], ADOM [49], GFLF [32], and ASNR [33]. To ensure a fair comparison, all competing algorithms are configured according to the parameter settings recommended in their original papers and evaluated on the same datasets. For our method, the guided filtering window size is set to [30 × 1], the smoothing parameter r=0.16, and the spectral entropy threshold scaling factor β=0.003. The MIRE [40] algorithm uses a Gaussian-weighted sliding midway histogram matching strategy. The Gaussian window’s standard deviation is automatically searched over the range [0, 8] with a step size 0.5. The MDBC [47] algorithm sets the regularization weight $m_{k}=0.1$ and the iterative step size Δt=0.1, with a maximum of 200 iterations. The TSF [36] method employs a striped band-stop filter in the frequency domain with a bandwidth K=2, followed by alternating 1 × 5 mean and Gaussian filtering in the spatial domain. The null-domain standard deviation is set to 1.2, and the number of iterations is capped at 10. For the ADOM [49] algorithm, the momentum coefficient is initialized to 1, the step-size adjustment coefficient δ=0.1, the penalty parameters $\rho_{1}$, $\rho_{2}$, and $\rho_{3}$ are empirically set to 5, and the regularization parameters $\lambda_{1}=\lambda_{2}=0.01$. The maximum number of iterations is 300, with a convergence threshold of $tol=1\times 10^{-4}$. The GFLF [32] algorithm is applied to OSU, LLVIP, KAIST, and Tendero’s datasets without image interception, and 1600 columns are intercepted for experiments in the long-wave infrared weekly scanning dataset. The horizontal filtering window of the GFLF algorithm is [8 × 1], ϵ=0.04, and the vertical filtering window is [1 × 100], ϵ=0.16. In the ASNR [33] algorithm, the guided filter window size is set to [5 × 5], and the smoothing coefficient is ϵ=0.01.

#### 3.5.2. Quantitative Testing of Simulated Datasets

To comprehensively evaluate the robustness and adaptability of each non-uniformity correction algorithm under different imaging resolutions and noise conditions, we construct simulated test sets using three publicly available infrared image datasets: OSU, KAIST, and LLVIP. For each dataset, 100 images are randomly selected. These datasets represent diverse application scenarios and resolutions:

(1) OSU Dataset [62]: Provided by Ohio State University, this dataset captures human activity scenes in natural outdoor environments. With a resolution of 240 × 320, it reflects low-resolution infrared imaging scenarios and is suitable for evaluating algorithm performance under small-scale conditions.
(2) KAIST Dataset [63]: Released by the Korea Advanced Institute of Science and Technology, this dataset includes city streets, parking lots, and varying illumination conditions across day, dusk, and night. The image resolution is 512 × 640, which provides a moderately complex environment for evaluating detail preservation and mid-scale stripe correction.
(3) LLVIP Dataset [64]: Developed by the University of Science and Technology of China, this dataset consists of indoor and outdoor scenes captured under low-light and nighttime conditions. The images include fine-grained thermal signatures from pedestrians, and the resolution is 1024 × 1280, making it ideal for high-resolution correction analysis.

Simulated noise is generated using the hybrid gain–bias–white noise model (Equation (22)) and the periodic noise model (Equation (23)). Equation (22) simulates column-based gain and bias distortions, while Equation (23) introduces stripe-like periodic interference along the scanning direction. To ensure disturbance significance and representativeness, the variance of all added noise components is no less than 0.01.

Table 3 lists five representative noise groups for each dataset, ranging from mild to severe non-uniformity. The parameter combinations include variations in gain variance $\sigma_{g}^{2}$, bias variance $\sigma_{b}^{2}$, white noise variance $\sigma_{white}^{2}$, periodic amplitude A, normalized frequency $f_{0}$, and phase offset ϕ. These synthetic conditions form a unified benchmark for evaluating correction performance under varying interference levels.

Each correction algorithm is tested on every image set under the corresponding noise group using these settings. The evaluation metrics include PSNR, SSIM, and roughness.

The results for the OSU dataset are presented in Table 4. Our method consistently achieves the highest PSNR and SSIM values across all noise groups. In contrast to MDBC and TSF, which tend to cause excessive smoothing or structural distortion, the proposed approach preserves finer textures while maintaining effective stripe suppression. For instance, in OSU-2, our method achieves a PSNR of 33.89 dB, outperforming MDBC (30.33 dB) and TSF (30.56 dB). Moreover, the resulting roughness is closer to the original noisy image, suggesting better structural preservation.

Table 5 presents results on the KAIST dataset, which features more complex scenes and high-frequency texture. Algorithms such as MDBC, ADOM, and ASNR perform poorly in the presence of cyclic and high-frequency noise. Notably, ASNR yields an SSIM of only 0.5774 in KAIST-5, suggesting significant detail loss. In contrast, our method maintains the best overall performance across all noise conditions, effectively balancing suppression and detail retention.

The results on the LLVIP dataset, shown in Table 6, further confirm the superiority of our algorithm under high-resolution and multi-source interference conditions. Traditional algorithms such as TSF and MDBC fail to effectively suppress fine-structured noise, especially in LLVIP-2 and LLVIP-5. Although GFLF and ASNR obtain moderate improvements, they still face limitations in managing complex noise patterns. In contrast, our method achieves PSNR values of 44.29 dB, 41.76 dB, and 37.05 dB in LLVIP-1, LLVIP-2, and LLVIP-4, respectively. Additionally, SSIM values exceed 0.97 across multiple groups, demonstrating the adaptability and robustness of our approach under extreme scenarios.

#### 3.5.3. Quantitative Testing on Real Datasets

To further validate the effectiveness of the proposed algorithm under real imaging conditions, we evaluate it on two real-world infrared datasets with different sources and scene characteristics. The first is a publicly available benchmark dataset (Tendero’s dataset), while the second is a self-collected long-wave infrared scanning dataset acquired in our laboratory. These datasets contain significant non-uniformity and structural complexity and are used to assess the robustness and effectiveness of the algorithms in scenarios without ideal reference images.

The first dataset is Tendero’s, which contains typical fixed-direction stripe noise and is widely used for benchmarking non-uniformity correction algorithms. All available images are used in the test, and the average values of all evaluation metrics are shown in Table 7.

Compared with other methods, our algorithm achieves the highest PSNR and SSIM while maintaining low roughness. MDBC and TSF show poor performance in GC and SSIM due to their reliance on global or frequency-domain statistical modeling and limited adaptability to local texture variations. ADOM effectively suppresses stripe components through energy reallocation, but its coupling of terms tends to cause over-smoothing in textured regions. In contrast, our method achieves stable suppression and structure preservation using spatial modeling and locally adaptive thresholding, resulting in the best performance across all indicators.

The second dataset is the long-wave infrared weekly scanning dataset, collected using a line-array scanning detector with a resolution of 1024 × 55,000. This dataset contains high-frequency fixed-pattern noise and complex background structures. For evaluation, 20 images are selected and cropped to 1024 × 8192. The average performance across these images is shown in Table 8.

As shown in Table 8, MIRE’s global sliding-window estimation strategy fails to address local gain fluctuations, resulting in residual stripe noise or structural artifacts. Although TSF partially suppresses cyclic interference, it frequently introduces boundary artifacts and struggles to accurately localize non-periodic distortions. ADOM and GFLF exhibit moderate performance but show significant deviations in roughness and NR, indicating challenges in preserving texture continuity. In contrast, our method effectively adapts to gain transitions and structural mutation regions, maintains low GC and roughness values, and consistently outperforms others in terms of PSNR and SSIM. These results confirm the strong generalization capability and robustness of the proposed approach in high-resolution infrared imaging scenarios.

#### 3.5.4. Qualitative Visualization Comparison

To further validate the proposed method’s visual performance, we conduct qualitative comparison experiments using three simulated noisy images (OSU-5, KAIST-3, and LLVIP-4) and two real infrared images (from Tendero’s dataset and a self-collected long-wave weekly infrared scanning dataset). These samples are selected to represent various interference patterns, structural complexities, and imaging resolutions.

In the simulated set, OSU-5 includes combined gain, bias, and periodic interference, making it suitable for evaluating overall denoising performance. KAIST-3 exhibits strong bias-enhanced stripes and low-contrast textures, emphasizing the need for structure preservation. LLVIP-4 comes from a high-resolution dataset with dominant periodic noise, which is ideal for testing frequency-domain robustness and edge detail recovery.

**Figure 11:**
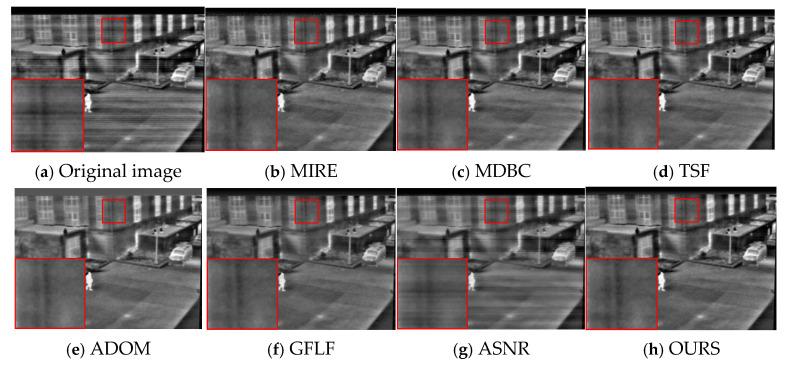
Visual comparison of correction results on OSU-5.

Figure 11 presents the visual results for OSU-5. In the low-contrast region marked by the red box, MIRE and MDBC exhibit limited suppression, with residual streaks and noticeable structural blur. TSF provides only weak stripe removal as periodic patterns remain visible. ADOM introduces over-smoothing due to global regularization, while GFLF preserves more structure but fails to eliminate periodic textures. ASNR effectively reduces interference but causes slight blurring of image details. In contrast, our method successfully eliminates stripe noise while preserving background textures and target clarity, demonstrating superior visual consistency and balance between denoising and detail retention.

**Figure 12:**
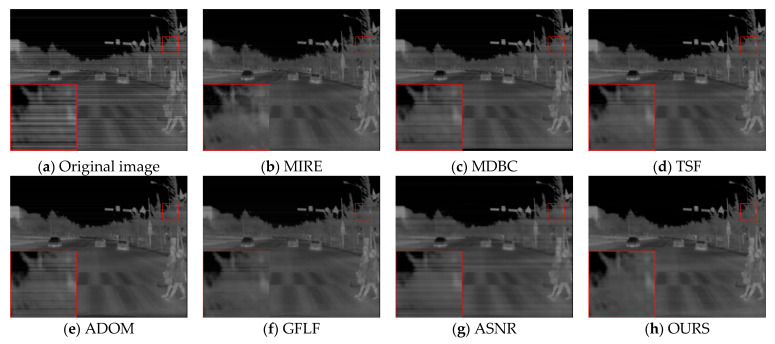
Visual comparison of correction results on KAIST-3.

As shown in Figure 12, for KAIST-3, MIRE and MDBC suffer from severe blurring and edge ambiguity. TSF suppresses some cyclic components but destroys structural texture, leading to an unnatural “oil painting” effect. ADOM results in over-compression and loss of weak texture, while GFLF and ASNR maintain moderate detail but leave residual stripe artifacts. Our method best preserves scene hierarchy and edge sharpness, balancing denoising and structure retention even in bias-enhanced scenarios.

**Figure 13:**
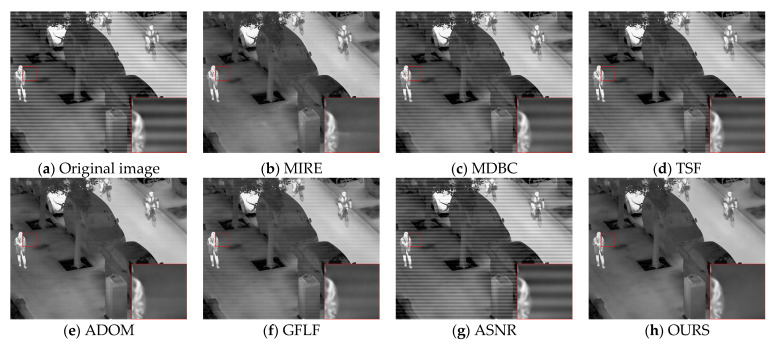
Visual comparison of correction results on LLVIP-4.

Figure 13 displays results for LLVIP-4. This high-resolution image with prominent periodic stripes poses challenges for frequency-domain methods. MIRE and MDBC fail to estimate stripe frequencies precisely, resulting in incomplete suppression. TSF focuses on band-stop filtering but induces over-smoothing. ADOM and GFLF preserve some texture but cannot entirely suppress periodic components. ASNR removes significant interference but introduces local luminance distortion. Our method shows strong frequency adaptability, suppressing periodic noise while retaining fine details and local contrast, especially in text and edge regions.

**Figure 14:**
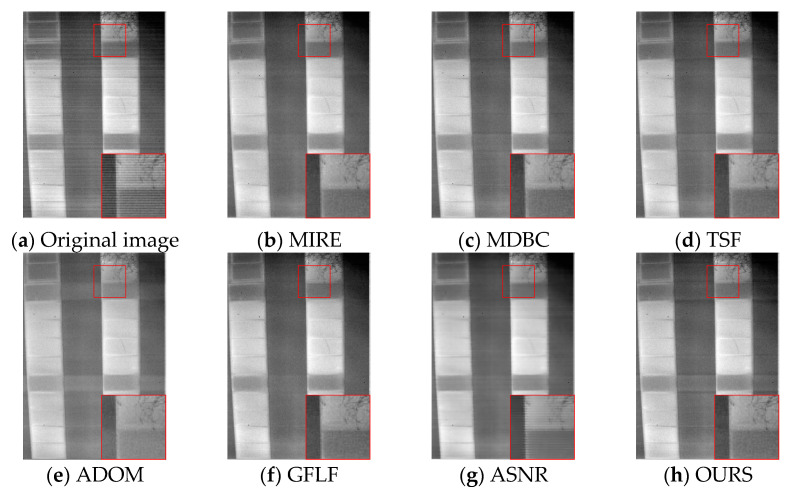
Visual comparison on Tendero’s dataset.

Figure 14 presents the result from Tendero’s dataset, which contains dense stripe patterns and weak structural texture for real images. Most methods can remove stripes to varying degrees, but MIRE, MDBC, and TSF compromise luminance uniformity. GFLF and ASNR achieve a better balance but may reduce detail sharpness. Our method effectively suppresses stripes while preserving edge clarity and natural brightness transitions, maintaining visual harmony and detail integrity in low-texture regions.

**Figure 15:**
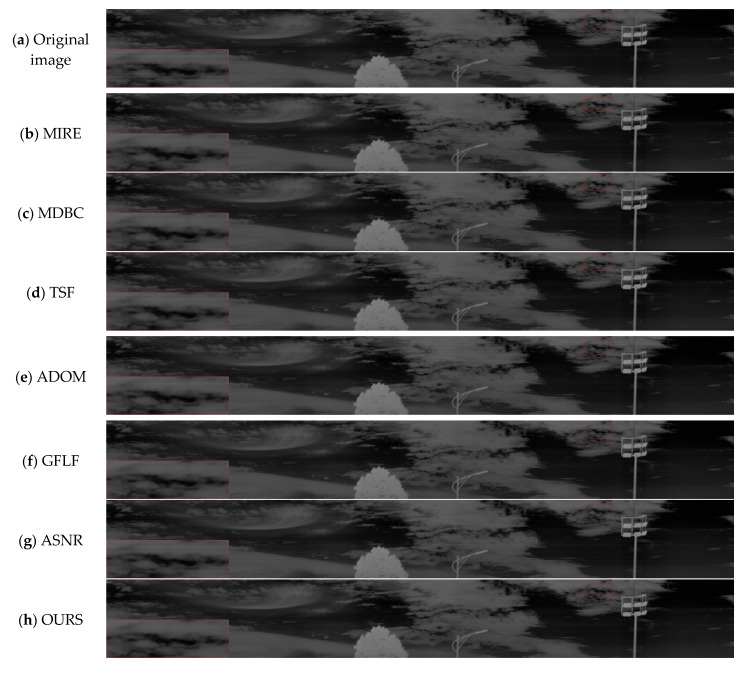
Visual comparison of long-wave infrared weekly scanning images.

Figure 15 shows the results of the long-wave infrared weekly scanning dataset, which our laboratory captured. The original image contains low-frequency periodic noise across the sky and cloud regions. TSF effectively suppresses interference but introduces uneven brightness transitions. MDBC yields smoother results but blurs detail. ADOM and GFLF retain some texture but suffer from over-suppression or artifacts. ASNR removes high-frequency noise but causes luminance inconsistency. Our method maintains brightness uniformity, clearly preserves cloud structures and pole contours, and avoids artifacts, demonstrating strong generalization to real long-format infrared scenes.

### 3.6. Runtime Comparison

To further evaluate the computational efficiency of the algorithms in practical applications, we measure the average runtime of seven algorithms when processing 30 real long-wave infrared weekly scanning images with a resolution of 1024 × 55,000. These high-resolution, long-width images place high demands on both algorithmic complexity and memory efficiency.

Table 9 summarizes the runtime comparison. Among traditional methods, MIRE exhibits the longest average runtime of 492.15 s, primarily due to its sliding-window median matching and global statistical fusion strategy, which require repeated block search and sorting across large-scale images. ADOM records an average runtime of 192.01 s as its ADMM-based framework involves iterative gradient computations and threshold updates, making it unsuitable for real-time applications. Although TSF features a relatively simple structure, its reliance on multiple FFT-based frequency-domain operations results in considerable runtime overhead for long-row images.

MDBC, GFLF, and ASNR achieve faster processing under certain conditions; however, they still incorporate region filtering, pyramid decomposition, or structure-tensor-based modeling. For example, ASNR attempts to balance edge preservation and interference suppression, but its multi-branch filtering leads to a runtime of 7.17 s.

In contrast, our method achieves the shortest average runtime of 0.1815 s, demonstrating excellent computational efficiency. The proposed algorithm adopts a lightweight structure comprising single-channel row-mean extraction, global guided filtering, and frequency-domain suppression. It requires only one global guided filtering operation and two FFTs—without involving image block search, iteration, or multi-scale reconstruction—thereby significantly reducing complexity.

Overall, the method ensures effective stripe removal and structural preservation while delivering outstanding runtime performance. Its low memory footprint, strong portability, and real-time capability make it particularly well suited for large-scale high-resolution column-scan image processing and embedded deployment in practical infrared imaging systems.

## 4. Discussion

Comprehensive experiments on three public datasets (OSU, KAIST, and LLVIP) and two real datasets (Tendero and a self-acquired long-wave infrared weekly scanning dataset) confirm that the proposed algorithm delivers superior performance in quantitative metrics and visual quality. Compared with recent state-of-the-art methods, it consistently achieves higher PSNR and SSIM, and lower roughness and GC, and demonstrates robustness across diverse noise patterns and resolutions. It notably achieves the shortest runtime among all tested methods, enabling real-time processing on large-scale images.

From a technical perspective, the strength of this algorithm stems from its three complementary components: (1) 1D row-mean modeling reduces data dimensionality while enhancing stripe orientation features; (2) MAD-driven adaptive guided filtering introduces spatial adaptivity, enabling fine-scale noise suppression without over smoothing; and (3) frequency-domain filtering with spectral entropy thresholding allows precise isolation of dominant interference components. These modules collectively form a spatial-frequency collaborative filtering framework that is both accurate and lightweight.

In addition to algorithmic effectiveness, the method is designed with engineering deployment in mind. Its reliance on only one guided filtering operation and two FFTs, without iterative refinement or patch-based processing, minimizes memory usage and computational load. This design ensures high portability and real-time applicability on hardware-constrained platforms such as embedded DSPs or FPGAs.

Nonetheless, certain limitations remain. The current method is optimized for horizontally oriented noise and does not yet generalize to arbitrary or oblique stripe patterns. Future work could explore direction-invariant models using log-Gabor filters or structure tensor-guided adaptive filtering. The algorithm is also passive to long-term detector drift; incorporating temporal frame sequences or on-chip calibration metadata may allow dynamic gain correction. Furthermore, integrating lightweight neural modules, such as temporal convolutional networks (TCNs) or spiking neural networks (SNNs), into the residual estimation stage may enhance accuracy while preserving interpretability and real-time performance.

## 5. Conclusions

This study proposes a lightweight and interpretable non-uniformity correction method for high-resolution infrared line-scan images affected by structural and periodic stripe noise. The technique achieves accurate stripe suppression by integrating 1D row-mean modeling, MAD-driven adaptive guided filtering, and spectral-entropy-constrained frequency masking while preserving texture and structural consistency.

Experimental validation across five datasets, including synthetic and real-world data, demonstrates that the proposed algorithm outperforms six representative state-of-the-art methods regarding PSNR, SSIM, roughness, GC, and NR. Moreover, it achieves sub-second runtime performance (0.18 s) on 1024 × 55,000 images, significantly surpassing traditional and optimization-based algorithms in computational efficiency.

Beyond its current design, the algorithm offers a scalable and robust framework for future enhancement. Potential extensions include handling arbitrary noise orientations, incorporating temporal modeling for long-term drift correction, and embedding explainable learning-based components for hybrid correction. The method holds strong potential for real-time infrared image correction in embedded, airborne, and satellite-based systems.

## Figures and Tables

**Table 1:** Five experimental configurations.

Combinatorial	DR	FM	ET	Clarification
B0 (Baseline)	×	×	×	Guided filtering using only large windows
B1 (B0 + DR)	√	×	×	Validating the local adaptive role of DR
B2 (B0 + FM)	×	√	×	Verification of periodic stripe suppression for FM
B3 (B0 + DR + FM)	√	√	×	Testing the complementarity of DR and FM
B4 (Full)	√	√	√	Full model

**Table 2:** Quantitative performance metrics of each module combination on real IR images.

Combinatorial	PSNR/(dB)	SSIM	Roughness	GC	NR
B0 (Baseline)	38.59	0.9164	0.0218	0.5478	0.999
B1 (B0 + DR)	38.88	0.9168	0.022	0.5488	1
B2 (B0 + FM)	39.49	0.9341	0.027	0.4925	0.999
B3 (B0 + DR + FM)	42.2	0.9652	0.0319	0.3558	1
B4 (Full)	42.44	0.9669	0.0324	0.3447	1.001

**Table 3:** Noise parameter settings for the OSU, KAIST, and LLVIP simulated datasets.

Noise Group	$\sigma_{g}^{2}$	$\sigma_{b}^{2}$	$\sigma_{white}^{2}$	A	$f_{0}$	ϕ	Characterization
OSU-1	0.01	0.01	---	---	---	---	Mild column bias and gain error
OSU-2	0.015	0.035	---	---	---	---	Offset dominant stripe structure enhancement
OSU-3	0.04	0.015	---	---	---	---	The gain of the dominant response varies significantly
OSU-4	---	---	---	0.05	0.08	π/3	Independent simulation of periodic disturbances
OSU-5	0.025	0.025	0.01	0.06	0.06	π/2	Stripe and periodic complex interference
KAIST-1	0.02	0.02	---	---	---	---	Moderately equilibrated non-homogeneous structures
KAIST-2	0.04	0.015	---	---	---	---	Gain dominance and texture perturbation
KAIST-3	0.025	0.045	---	---	---	---	Bias enhancement with distinctive streaks
KAIST-4	---	---	---	0.07	0.07	π/4	Simulation of purely periodic mains frequency interference
KAIST-5	0.03	0.03	0.01	0.08	0.05	π/2	Structural streaks and cyclic coupling
LLVIP-1	0.02	0.015	---	---	---	---	Slightly non-uniform structure at high resolution
LLVIP-2	0.035	0.025	---	---	---	---	Gain dominates band microstructure texture interference
LLVIP-3	0.03	0.04	0.02	---	---	---	Bias enhancement with a bit of white noise
LLVIP-4	---	---	---	0.09	0.04	π/2	High-resolution periodic stripe jitter characteristics
LLVIP-5	0.04	0.04	0.01	0.12	0.03	π	Multi-source joint extreme interference simulation

**Table 4:** Quantitative results on the OSU simulated dataset.

Simulated Image Data	Metric	Noise	MIRE	MDBC	TSF	ADOM	GFLF	ASNR	OURS
OSU-1	PSNR	---	35.62	32.36	31.18	22.46	33.27	29.11	36.39
	SSIM	---	0.9641	0.9381	0.934	0.8782	0.9397	0.8895	0.9686
	Roughness	0.1275	0.1179	0.1179	0.1164	0.1059	0.1142	0.0782	0.1202
OSU-2	PSNR	---	31.68	30.33	30.56	22.34	32.21	28.15	33.89
	SSIM	---	0.9211	0.9186	0.9208	0.8512	0.9274	0.8199	0.9361
	Roughness	0.1898	0.1229	0.1205	0.1175	0.1032	0.1184	0.0956	0.1273
OSU-3	PSNR	---	33.06	31.73	30.77	22.34	32.57	28.59	34.83
	SSIM	---	0.9419	0.9243	0.9221	0.8551	0.9279	0.8567	0.9501
	Roughness	0.1531	0.1233	0.1222	0.1202	0.1087	0.1188	0.0864	0.1274
OSU-4	PSNR	---	29.49	30.51	30.66	22.08	31.66	28.24	32.18
	SSIM	---	0.9047	0.9045	0.9239	0.8422	0.9272	0.8294	0.9305
	Roughness	0.1311	0.1237	0.1184	0.1156	0.1031	0.1135	0.091	0.1288
	PSNR	---	28.3	29.23	29.35	22.42	31.18	26.51	31.82
OSU-5	SSIM	---	0.8592	0.8648	0.8768	0.8142	0.8918	0.7661	0.9085
	Roughness	0.1836	0.1428	0.1416	0.139	0.1164	0.1374	0.1135	0.1453

**Table 5:** Quantitative results on the KAIST simulated dataset.

Simulated Image Data	Metric	Noise	MIRE	MDBC	TSF	ADOM	GFLF	ASNR	OURS
KAIST-1	PSNR	---	40.87	34.64	41.45	35.24	41.58	39.9	42.25
	SSIM	---	0.9271	0.9112	0.9299	0.8792	0.9274	0.9153	0.9302
	Roughness	0.2204	0.0631	0.0825	0.0705	0.0591	0.0676	0.0607	0.091
KAIST-2	PSNR	---	41.73	34.8	42.27	39.26	42.28	41.02	42.64
	SSIM	---	0.9393	0.9194	0.9405	0.9178	0.9382	0.9327	0.9431
	Roughness	0.1875	0.0645	0.084	0.0727	0.0695	0.0695	0.0561	0.102
KAIST-3	PSNR	---	36.84	33.1	36.27	32.54	36.9	34.81	37.27
	SSIM	---	0.8687	0.8321	0.8638	0.7998	0.8623	0.8243	0.8755
	Roughness	0.434	0.0704	0.1076	0.0866	0.0797	0.0877	0.0871	0.1119
KAIST-4	PSNR	---	38.56	31.89	36.8	30.17	38.56	29.81	39.57
	SSIM	---	0.8757	0.7785	0.8488	0.7423	0.8753	0.6493	0.8938
	Roughness	0.1785	0.0518	0.0916	0.0686	0.0901	0.0563	0.1232	0.132
KAIST-5	PSNR	---	34.51	27.88	30.47	32.25	34.26	27.18	36.6
	SSIM	---	0.7862	0.6646	0.7098	0.7447	0.7817	0.5774	0.8548
	Roughness	0.3886	0.192	0.2235	0.2097	0.2215	0.1985	0.2194	0.2406

**Table 6:** Quantitative results on the LLVIP simulated dataset.

Simulated Image Data	Metric	Noise	MIRE	MDBC	TSF	ADOM	GFLF	ASNR	OURS
LLVIP-1	PSNR	---	41.45	40.33	42.47	32.36	43.73	39.88	44.29
	SSIM	---	0.9804	0.9621	0.9726	0.8917	0.9798	0.9706	0.9827
	Roughness	0.0675	0.028	0.0356	0.033	0.0326	0.0307	0.0276	0.0378
LLVIP-2	PSNR	---	39.32	36.45	39.22	32.07	41.04	37.42	41.76
	SSIM	---	0.9738	0.9302	0.9521	0.8807	0.9682	0.9474	0.9747
	Roughness	0.1004	0.0292	0.0424	0.038	0.0365	0.0344	0.0342	0.0478
LLVIP-3	PSNR	---	33.53	32.54	33.9	31.13	34.5	33.8	35.71
	SSIM	---	0.8055	0.7891	0.8051	0.7909	0.8119	0.8254	0.847
	Roughness	0.1613	0.0785	0.0822	0.08	0.0702	0.079	0.0565	0.0827
LLVIP-4	PSNR	---	36.1	27.91	28.17	26.24	34.74	25.17	37.05
	SSIM	---	0.9625	0.8207	0.0832	0.7776	0.9527	0.723	0.9712
	Roughness	0.0509	0.0272	0.0392	0.0381	0.0416	0.0278	0.0454	0.047
	PSNR	---	28.3	29.23	29.35	22.42	31.18	26.51	31.82
LLVIP-5	SSIM	---	0.8592	0.8648	0.8768	0.8142	0.8918	0.7661	0.9085
	Roughness	0.1836	0.1428	0.1416	0.139	0.1164	0.1374	0.1135	0.1453

**Table 7:** Quantitative results on Tendero’s dataset.

Real Image Data	Metric	Noise	MIRE	MDBC	TSF	ADOM	GFLF	ASNR	OURS
Tendero’s data	PSNR	---	25.63	21.71	25.65	23.72	27.84	28.34	29.74
	SSIM	---	0.6043	0.6219	0.6048	0.5761	0.7811	0.745	0.8296
	Roughness	0.3357	0.1419	0.1515	0.1429	0.1298	0.2118	0.1105	0.2291
	GC	---	0.5014	0.418	0.4287	0.4204	0.3663	0.4974	0.3322
	NR	---	1.041	1.0318	1.042	1.058	1.0344	1.0428	1.0671

**Table 8:** Quantitative results on the long-wave infrared weekly scanning dataset.

Real Image Data	Metric	Noise	MIRE	MDBC	TSF	ADOM	GFLF	ASNR	OURS
Long-wave infrared weekly scanning dataset	PSNR	---	36.55	37.05	36.96	30.85	36.63	37.27	37.78
	SSIM	---	0.8372	0.8601	0.844	0.807	0.8415	0.8721	0.8784
	Roughness	0.0393	0.0305	0.0361	0.0337	0.0215	0.0315	0.0189	0.0372
	GC	---	0.8638	0.7728	0.8245	0.8462	0.8286	0.8428	0.5748
	NR	---	1.0097	1.0115	1.0089	1.0367	1.0125	1.0092	1.0417

**Table 9:** Comparison of the time complexity of different algorithms.

Algorithms	MIRE	MDBC	TFS	ADOM	GFLF	ASNR	OURS
Time/s	492.1527	0.2744	5.9356	192.0058	1.5345	7.1714	0.1815

## Data Availability

An infrared long-wave cooled linear scan detector generated the real infrared image dataset. It is not a public dataset. The publicly available dataset OSU was analyzed in this study and can be found here: http://vcipl-okstate.org/pbvs/bench/Data/01/download.html, accessed on 20 April 2025. The publicly available dataset KAIST was analyzed in this study and can be found here: https://soonminhwang.github.io/rgbt-ped-detection/, accessed on 20 April 2025.The publicly available dataset LLVIP was analyzed in this study and found here: https://github.com/bupt-ai-cz/LLVIP, 20 April 2025. The publicly available Tendero dataset was analyzed in this study and can be found here: https://ipolcore.ipol.im/demo/clientApp/demo.html?id=129, accessed on 22 April 2025.
